# Supplementary material for: Definitive Endodermal Cells Supply an in vitro Source of Mesenchymal Stem/Stromal Cells
Source: Commun Biol. 2023 May 1;6:476. doi: 10.1038/s42003-023-04810-5 (PMC10151361; doi:10.1038/s42003-023-04810-5)
Supplement: Supplementary file 2 — Supplementary Information [file 42003_2023_4810_MOESM2_ESM.pdf]

Supplementary Figure 1.

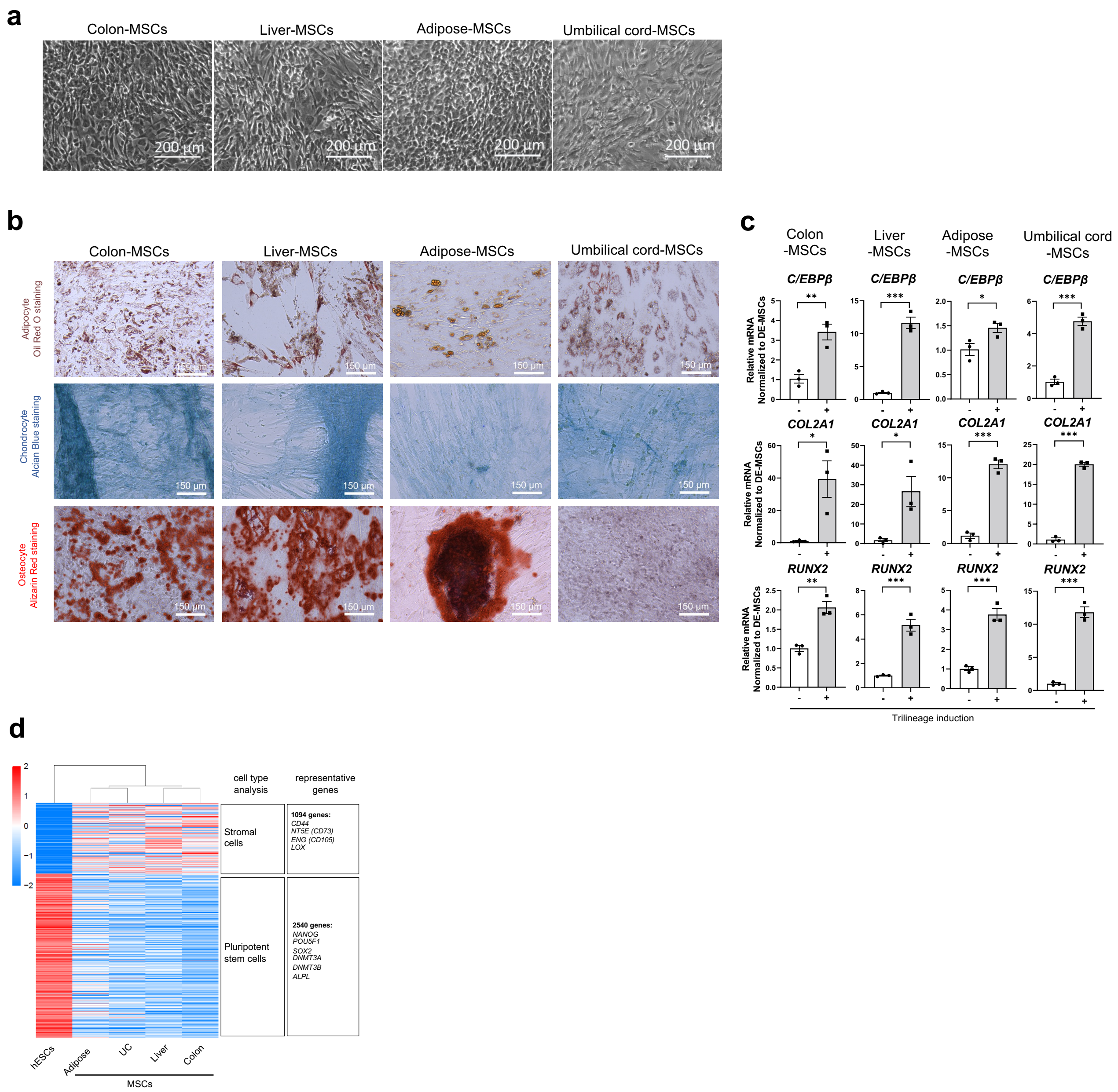

Supplementary Figure 1. Organ-specific transcriptomes are observed in MSCs isolated from different embryonic lineages. a Morphology of colon-, liver-, adipose- and umbilical cord-MSCs, scale bar = 200 µm. **b** Colon, liver, adipose and umbilical cord-MSCs can be induced into adipocytes (stained with Oil Red O), chondrocytes (stained with Alcian Blue) and osteocytes (stained with Alizarin Red). Scale bar = 150 µm. **c** RT-qPCR analysis of adipogenic (*C/EBPβ*), chondrogenic (*COL2A1*) and osteogenic (*RUNX2*) marker genes (n = 3), $*p < 0.05$, $**p < 0.01$, $***p < 0.001$. **d** Heatmap of DEG in hESCs, colon-, liver-, adipose- and umbilical cord-MSCs and analysis of cell type via Enrichr database.

Supplemental Figure 2.

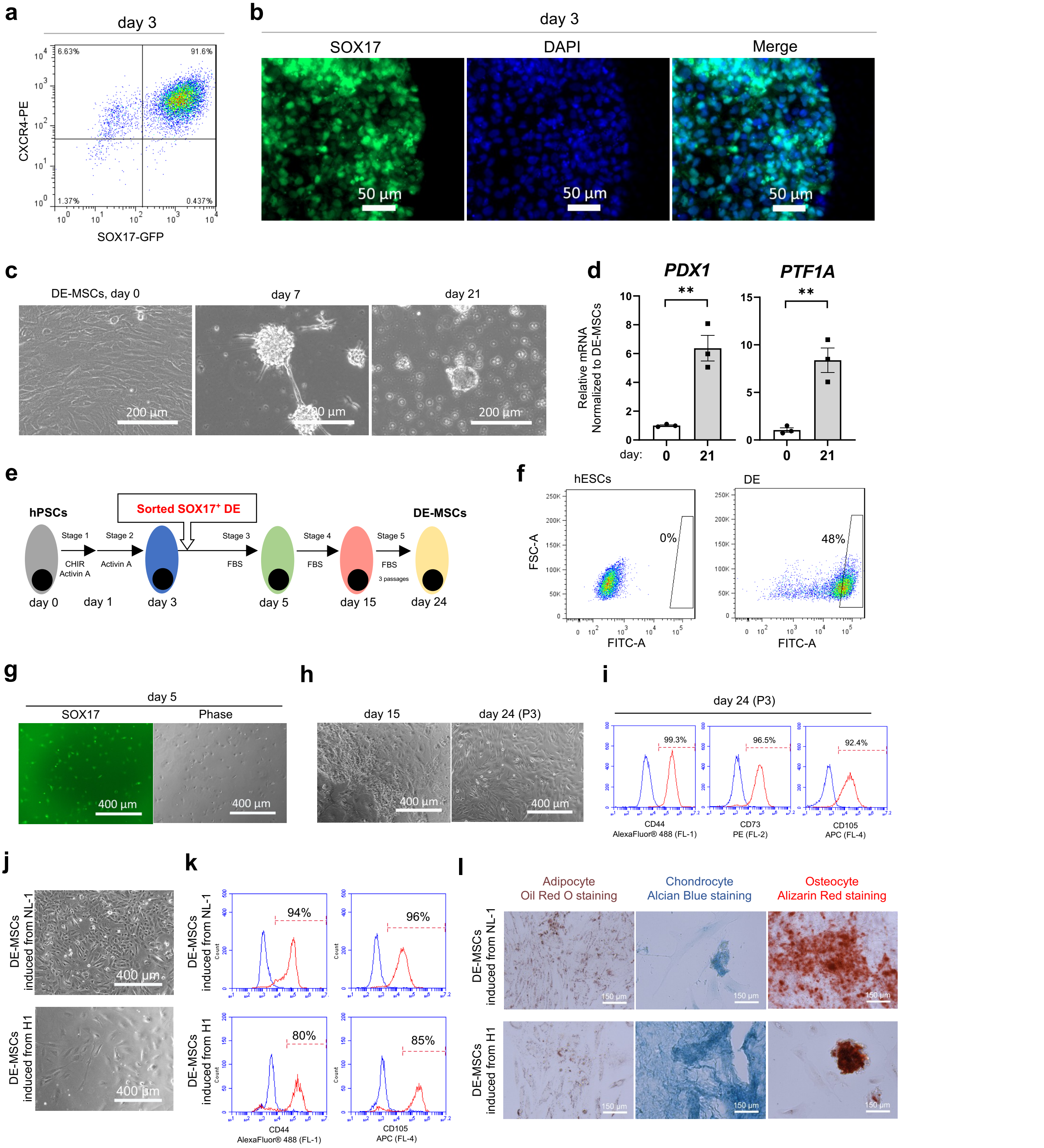

Supplementary Figure 2. MSCs are derived from hESCs and iPSCs via definitive endoderm progenitors. a Flow cytometry assay showing the percentage of CXCR4 / SOX17 double positive cells on day 3, undifferentiated hESCs were used as gating strategy. **b** Fluorescence images showing SOX17 positive DE progenitors on day 3. **c** DE-MSCs form islet-like spatial structures on day 21. **d** mRNA levels of pancreatic progenitor marker genes *PDX1* and *PTF1A* were upregulated on day 21 (n = 3). **$p < 0.01$. **e** Schematic diagram indicating the purification of SOX17$^+$ DE progenitors on day 3 and induction of DE-MSCs from purified SOX17$^+$ DE. **f** SOX17$^+$ DE progenitors were purified and seeded onto Matrigel-coated 24-well plate (50,000 cells per well) in MSC medium, undifferentiated hESCs were used as gating strategy. **g** Fluorescence images showing that purified cells were all SOX17-positive on day 5. Scale bar = 400 µm. **h** Phase contrast images showing the heterogeneous population on day 15 in FBS conditions, and spindle like MSC morphology was observed on day 24. Scale bar = 400 µm. **i** Flow cytometry images showing that DE-MSCs induced from purified DE were positive for CD44, CD73 and CD105 on day 24, blue line represented IgG isotype control for gating strategy, red line represented the percentage of CD44$^+$, CD73$^+$ and CD105$^+$ cells. **j** Morphology of DE-MSCs induced from H1 hESCs and NL-1 hiPSCs. **k** Flow cytometry assay showing that DE-MSCs from H1 hESCs and NL-1 hiPSC lines were CD44 and CD105 positive, blue line represented IgG isotype control for gating strategy, red line represented the percentage of CD44$^+$ and CD105$^+$ cells. **l** DE-MSCs induced from H1 or NL-1 were induced into adipocytes (stained with Oil Red O), chondrocytes (stained with Alcian Blue) and osteocytes (stained with Alizarin Red). Scale bar = 150 µm.

Supplemental Figure 3.

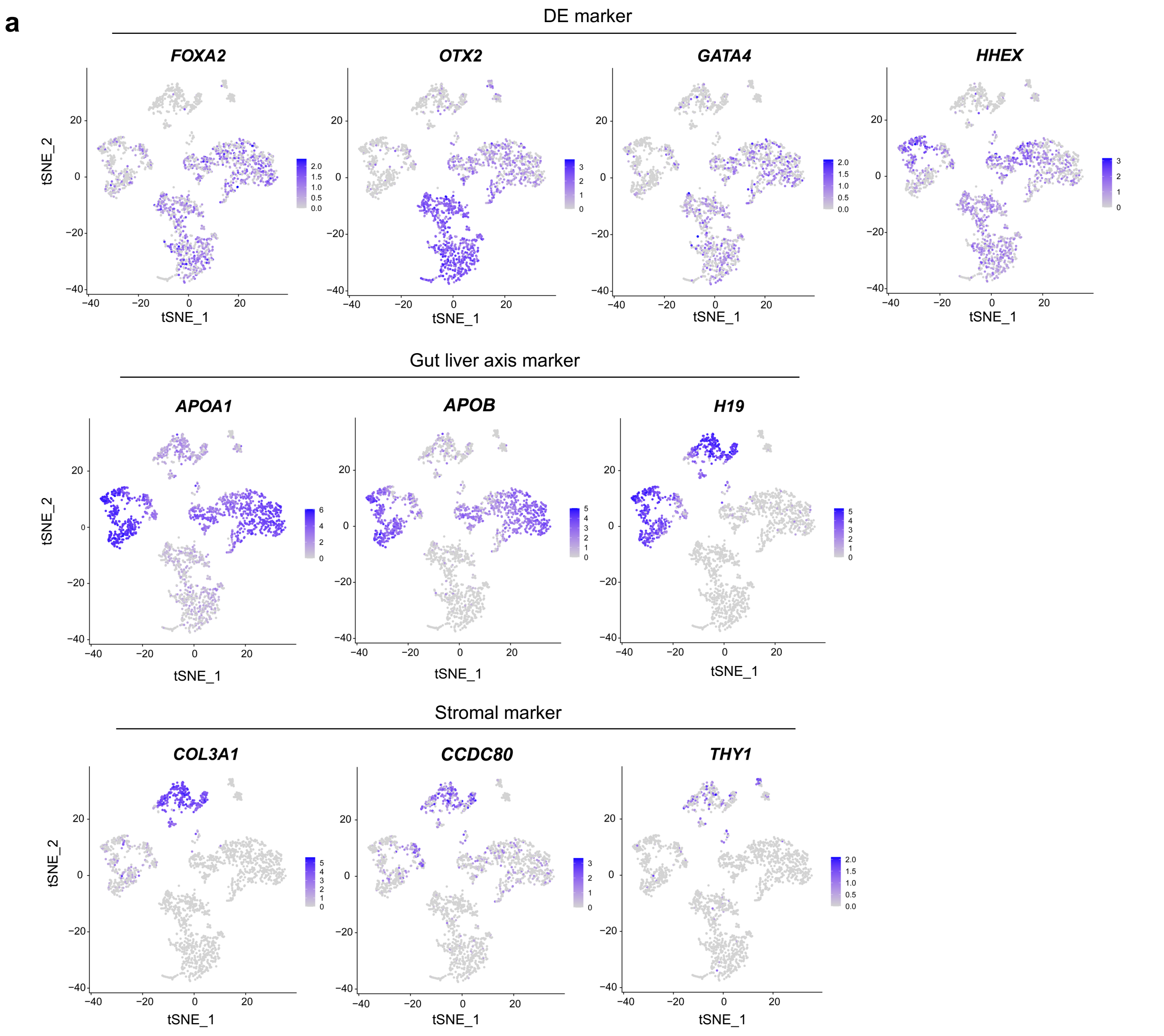

Supplementary Figure 3. scRNA-seq reveals the emergence of DE-MSCs *in vitro*. a tSNE projection of DE markers (*FOXA2*, *OTX2*, *GATA4* and *HHEX*) expression, gut liver axis markers (*APOA1*, *APOB* and *H19*) expression and stromal markers (*COL3A1*, *CCDC80* and *THY1*) expression on day 3 (DE), day 5 and day 15 clusters at single cell resolution, showing that cells in cluster 2 of day 15 sample expressed stromal marker genes and gut liver axis marker genes.

Supplemental Figure 4.

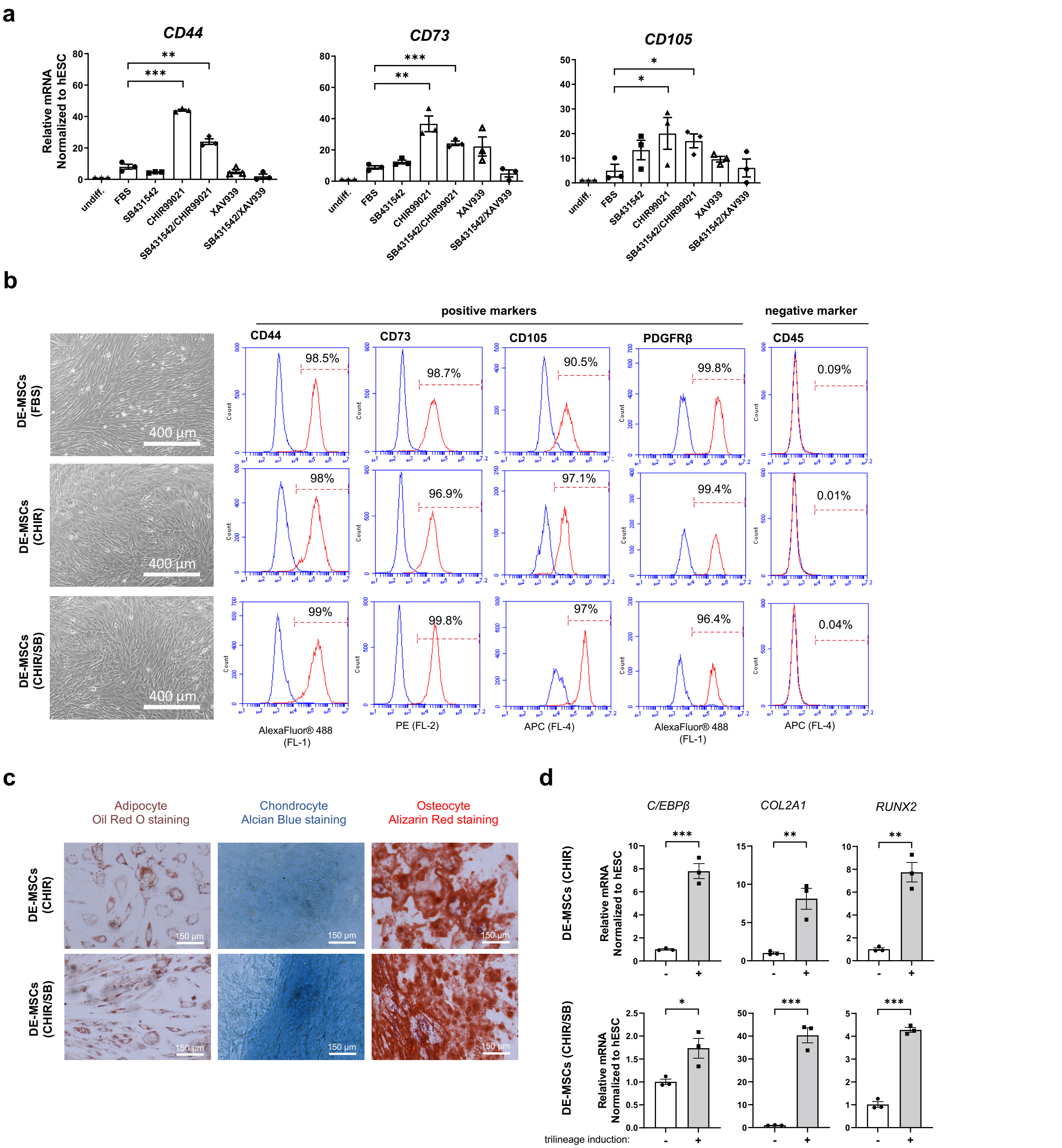

Supplementary Figure 4. Signal modulations affect DE-MSC induction. a RT-qPCR analysis showing the influence of short term (2 days) treatment of CHIR99021, SB431542, XAV939 and their combination on the expression of *SOX17*, *CD44*, *NT5E (CD73)* and *ENG (CD105)* on day 15 (n = 3). *p < 0.05, **p < 0.01, ***p < 0.001. **b** Left panel, morphology of DE-MSCs (FBS) (DE treated for additional 2 days under FBS condition), DE-MSCs (CHIR) (DE treated with CHIR99021 for additional 2 days under FBS condition), and DE-MSCs (CHIR/SB) (DE treated with CHIR99021 and SB431542 for additional 2 days under FBS condition) with typical spindle MSC shape. Scale bar = 400 µm. Right panel, flow cytometry showing that those cells were CD44, CD73, CD105 and PDGFRβ positive, but CD45 negative. Blue line represented IgG isotype control for gating strategy, red line represented the percentage of $CD44^+$, $CD73^+$, $CD105^+$, $PDGFR\beta^+$ and $CD45^+$ cells. **c** DE-MSCs (CHIR) and DE-MSCs (CHIR/SB) were induced into adipocytes (stained with Oil Red O), chondrocytes (stained with Alcian Blue) and osteocytes (stained with Alizarin Red). Scale bar = 150 µm. **d** RT-qPCR analysis of adipogenic (*C/EBPβ*), chondrogenic (*COL2A1*) and osteogenic (*RUNX2*) marker genes (n = 3), *p < 0.05, **p < 0.01, ***p < 0.001.

Supplemental Figure 5.

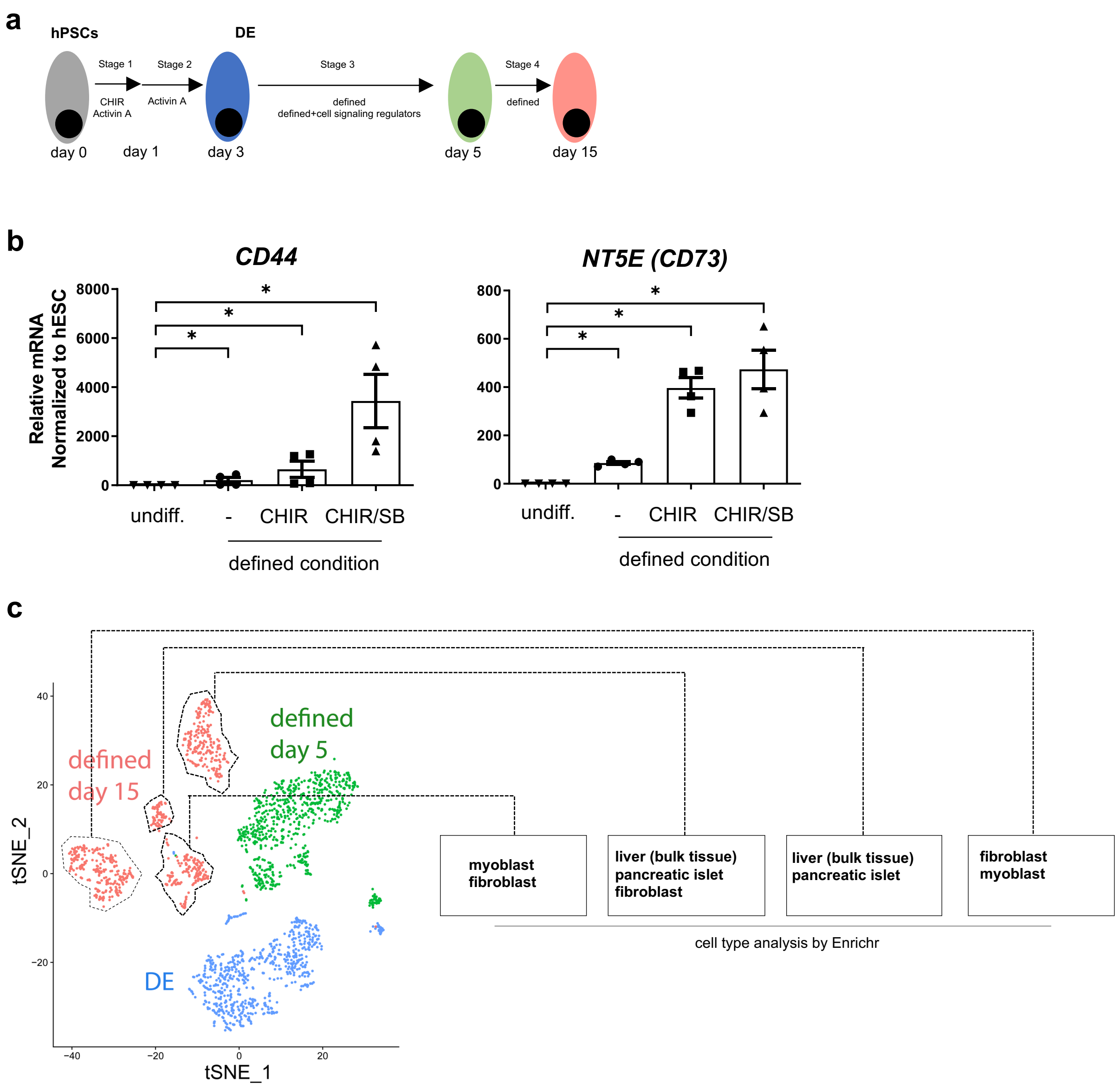

Supplementary Figure 5. DE-MSCs are induced in serum-free conditions. a Schematic DE-MSC induction strategy in serum-free conditions. **b** RT-qPCR analysis showing that the expression of *CD44* and *NT5E (CD73)* was elevated by CHIR99021 or CHIR99021+SB431542 treatment in serum-free medium. **c** tSNE projection of day 3 (DE), day 5 and day 15 samples under serum-free conditions, and cell type analysis via Enrichr database for the different clusters of day 15 sample, indicating MSCs originated from definitive endoderm under serum free condition were enriched with organ-specific gene expression.

Supplemental Figure 6.

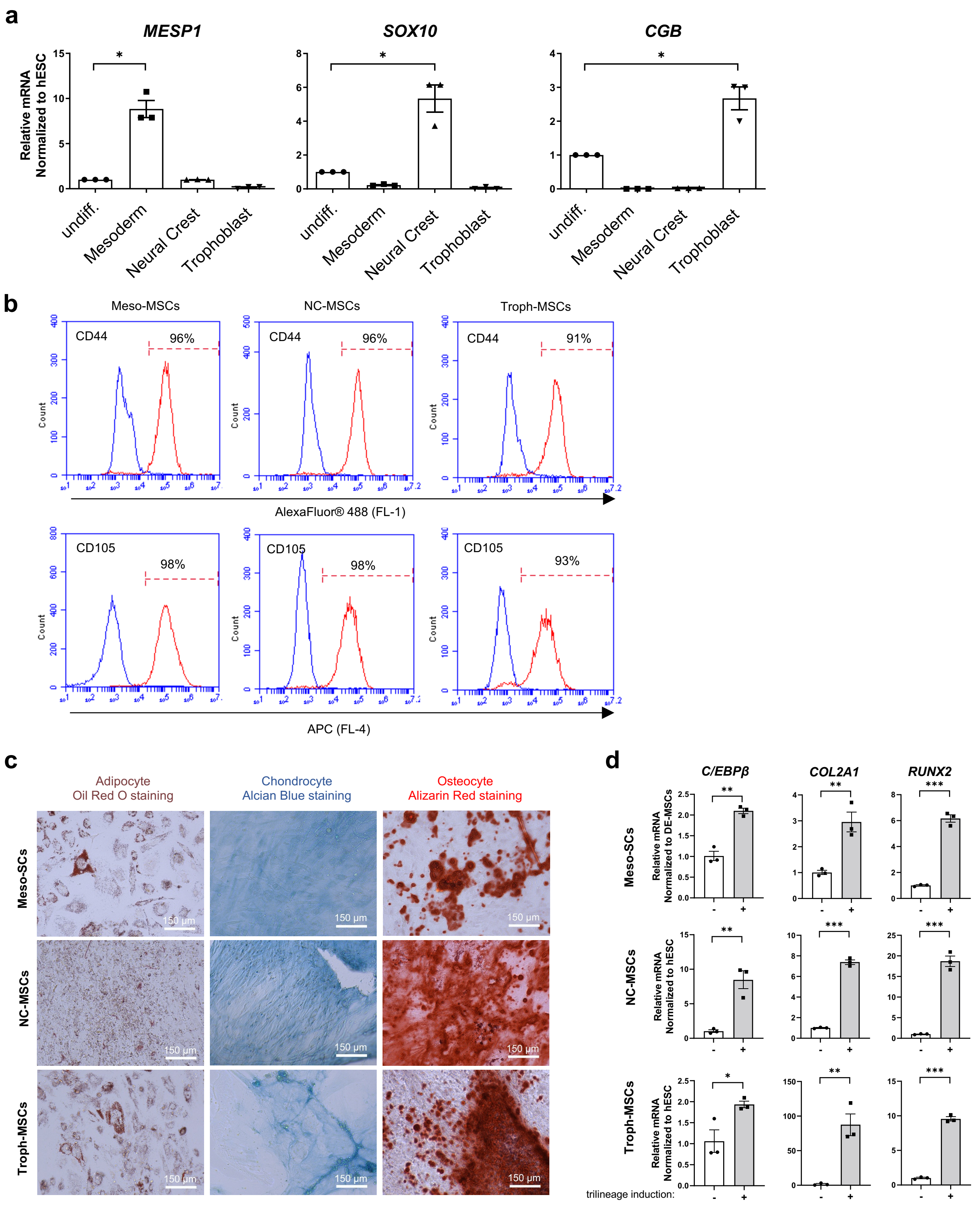

Supplementary Figure 6. Generation of mesoderm-, neural crest-, trophoblast-MSCs. **a** RT-qPCR assay showing that mesoderm marker *MESP1*, ectoderm marker *SOX10* and extraembryonic marker *CGB* were expressed in mesoderm progenitors, neural crest progenitors and trophoblast progenitors, respectively. **b** Flow cytometry assay showing the expression of CD44 and CD105 in meso-, NC-, troph-MSCs. Blue line represented IgG isotype control for gating strategy, red line represented the percentage of CD44$^+$ and CD105$^+$ cells. **c** Meso-, NC- and Tropho-MSCs were induced into adipocytes (stained with Oil Red O), chondrocytes (stained with Alcian Blue) and osteocytes (stained with Alizarin Red). Scale bar = 150 μm. **d** RT-qPCR analysis of adipogenic (*C/EBPβ*), chondrogenic (*COL2A1*) and osteogenic (*RUNX2*) marker genes (n = 3), *p < 0.05, **p < 0.01, ***p < 0.001.

Supplemental Figure 7.

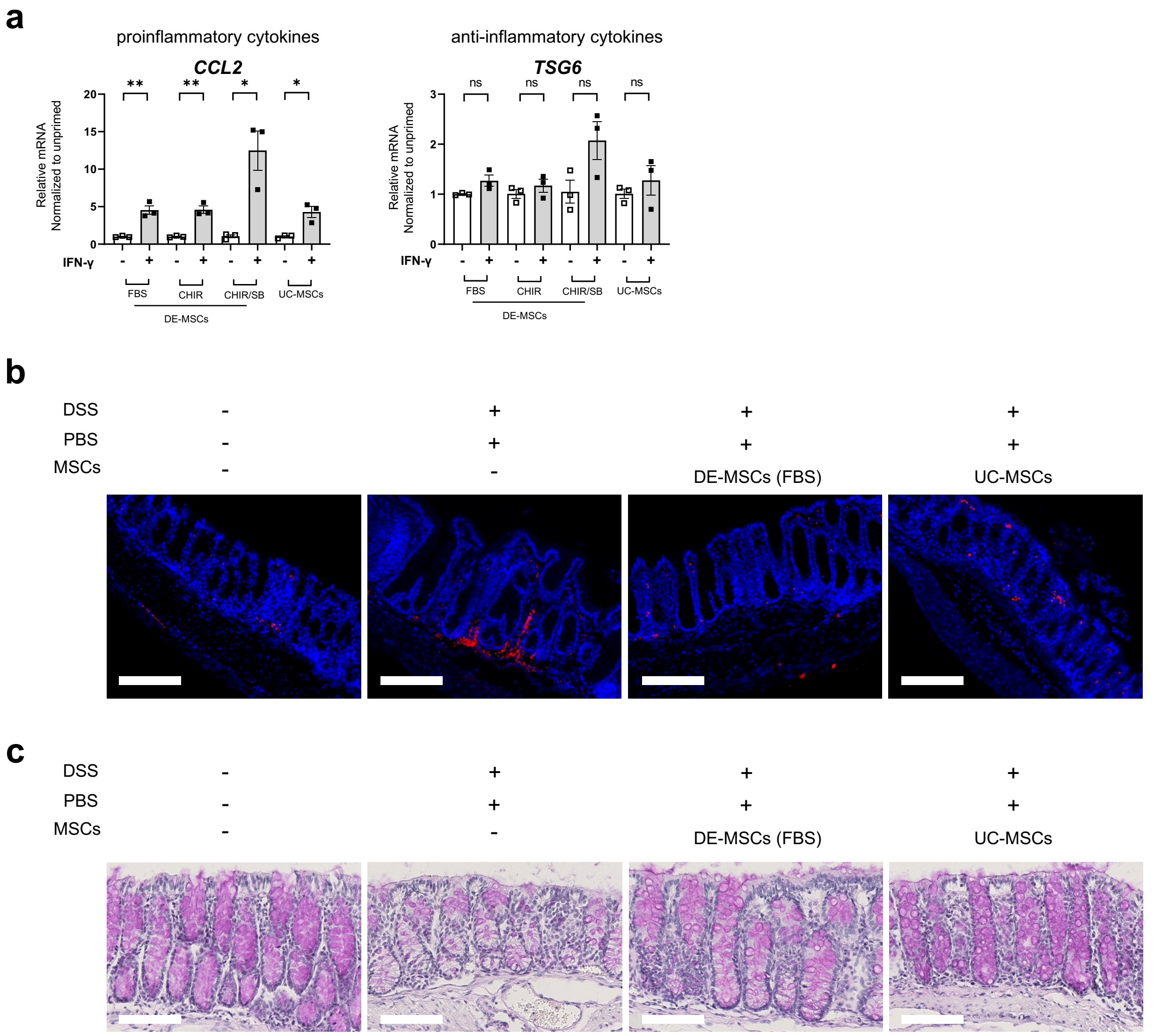

Supplementary Figure 7. DE-MSCs modulate inflammatory responses in cell culture and mouse model. **a** mRNA levels of proinflammatory cytokine *CCL2* and anti-inflammatory cytokine *TSG6* were analyzed by RT-qPCR assay in DE-MSCs (FBS), DE-MSCs (CHIR), DE-MSCs (CHIR/SB) and UC-MSCs that were exposed to IFN-γ (n = 3). *p < 0.05, **p < 0.01 and ns is non-significant. **b** Immunostaining for $CD8^+$ cytotoxic T cells (red) in distal colon sections from healthy mice and DSS+PBS, DSS+ PBS+DE-MSCs (FBS), DSS+PBS+UC-MSCs groups, nucleus were stained with DAPI (blue), scale bar = 100 µm. **c** AB/PAS staining of mucin-producing goblet cells of distal colon sections from healthy mice and DSS+PBS, DSS+ PBS+DE-MSCs (FBS), DSS+PBS+UC-MSCs groups, scale bar = 100 µm.

Supplementary Table 1. Chemicals and recombinant proteins

**Chemicals and recombinant proteins**	**Brand**	**Cat No.**
Penicillin/streptomycin	ThermoFisher	Cat.# 15140122
Transferrin	Sigma	Cat.# T0665
Ascorbic acid	Sigma	Cat.# A8960
Insulin	Sigma	Cat.# I9278
Recombinant human TGFβ	R&D	Cat.# 240-B-001MG/CF
Recombinant human Activin A	R&D	Cat.# 338-AC-050/CF
CHIR-99021 (CT99021) HCl	Selleck	Cat.# S2924
XAV939	Selleck	Cat.# S1180
SB 431542	Selleck	Cat.# S1067
Matrigel®	Corning	Cat.# 354230
MEM α, nucleosides	Gibco	Cat.# 12571063
DMEM/F-12, HEPES	Gibco	Cat.# 11330032
MEM Non-Essential Amino Acids Solution	Gibco	Cat.# 11140050
GlutaMAX^{TM}-I	Gibco	Cat.# A1286001
Fetal Bovine Serum	Gibco	Cat.# 10437028

Supplementary Table 2. Primers used for RT-qPCR

mRNA Transcript	Forward primer	Reverse primer
SOX17	GGATCAGGGACCTGTCACAC	CGCACGGAATTTGAACAGTA
FOXA2	GCATTCCCAATCTTGACACGGTGA	GCCCTTGCAGCCAGAATACACATT
CDX2	GGAACCTGTGCGAGTGGAT	TCCGTGTACACCACTCGATATT
HOXC5	AGAGCCCCAATATCCCTGC	CGGTGGGAAAGTGATGCTT
HHEX	CACCCGACGCCCTTTTACAT	GAAGGCTGGATGGATCGGC
MESP1	GAAGTGGTTCCTTGGCAGAC	TCCTGCTTGCCTCAAAGTGT
SOX10	TGCAGCACAAGAAAGACCAC	GCCACATCAAAGGTCTCCAT
CGB	TCACCGTCAACACCACCATC	AGAGTGCACATTGACAGCTG
CD44	TGGCACCCGCTATGTCGAG	GTAGCAGGGATTCTGTCTG
NT5E (CD73)	CTCCTCTCAATCATGCCGCT	CATCAATGGGCGACCGGATA
ENG (CD105)	TTGTGCAGGTCAGAGTGTCC	GCCGGTTTTGGGTATGGGTA
NGFR (CD271)	CCTACGGCTACTACCAGGATG	CACACGGTGTTCTGCTTGT
IL-6	ACTCACCTCTTCAGAACGAATTG	CCATCTTTGGAAGGTTCAGGTTG
IL-8	TTTTGCCAAGGAGTGCTAAAGA	AACCCTCTGCACCCAGTTTTC
CCL2	CAGCCAGATGCAATCAATGCC	TGGAATCCTGAACCCACTTCT
IDO1	GCCAGCTTCGAGAAAGAGTTG	ATCCCAGAACTAGACGTGCAA
PD-L1	TGGCATTTGCTGAACGCATTT	TGCAGCCAGGTCTAATTGTTTT
TSG6	TTTCTCTTGCTATGGGAAGACAC	GAGCTTGTATTTGCCAGACCG
FABP4	ACTGGGCCAGGAATTTGACG	CTCGTGGAAGTGACGCCTT
CEBPB	CTTCAGCCCGTACCTGGAG	GGAGAGGAAGTCGTGGTGC
RUNX2	AGCCCTCGGAGAGGTACCA	CGGAGCTCAGCAGAATAATTTTC
OCN	CACTCCTCGCCCTATTGGC	CCCTCCTGCTTGGACACAAAG
COL1A1	GAGGGCCAAGACGAAGACATC	CAGATCACGTCATCGCACAAC
COL2A1	TGGACGATCAGGCGAAACC	GCTGCGGATGCTCTCAATCT
PDX1	ATCTCCCCATACGAAGTGCC	CGTGAGCTTTGGTGGATTTCAT
GAPDH	GTGGACCTGACCTGCCGTCT	GGAGGAGTGGGTGTCGCTGT
TBP	CCACTCACAGACTCTCACAAC	CTGCGGTACAATCCCAGAACT
