# Supplementary material for: Definitive Endodermal Cells Supply an in vitro Source of Mesenchymal Stem/Stromal Cells
Source: Commun Biol. 2023 May 1;6:476. doi: 10.1038/s42003-023-04810-5 (PMC10151361; doi:10.1038/s42003-023-04810-5)
Supplement: Supplementary file 3 — Reporting summary [file 42003_2023_4810_MOESM3_ESM.pdf]

nature portfolio

Corresponding author(s): DBPR, COMMSBIO-22-0585A

Last updated by author(s): Dec 14, 2022

Reporting Summary

Nature Portfolio wishes to improve the reproducibility of the work that we publish. This form provides structure for consistency and transparency in reporting. For further information on Nature Portfolio policies, see our Editorial Policies and the Editorial Policy Checklist.

Statistics

For all statistical analyses, confirm that the following items are present in the figure legend, table legend, main text, or Methods section.

n/a	Confirmed	
☐	☒	The exact sample size (*n*) for each experimental group/condition, given as a discrete number and unit of measurement
☐	☒	A statement on whether measurements were taken from distinct samples or whether the same sample was measured repeatedly
☐	☒	The statistical test(s) used AND whether they are one- or two-sided<br>*Only common tests should be described solely by name; describe more complex techniques in the Methods section.*
☒	☐	A description of all covariates tested
☒	☐	A description of any assumptions or corrections, such as tests of normality and adjustment for multiple comparisons
☐	☒	A full description of the statistical parameters including central tendency (e.g. means) or other basic estimates (e.g. regression coefficient) AND variation (e.g. standard deviation) or associated estimates of uncertainty (e.g. confidence intervals)
☐	☒	For null hypothesis testing, the test statistic (e.g. *F*, *t*, *r*) with confidence intervals, effect sizes, degrees of freedom and *P* value noted<br>*Give P values as exact values whenever suitable.*
☒	☐	For Bayesian analysis, information on the choice of priors and Markov chain Monte Carlo settings
☒	☐	For hierarchical and complex designs, identification of the appropriate level for tests and full reporting of outcomes
☒	☐	Estimates of effect sizes (e.g. Cohen's *d*, Pearson's *r*), indicating how they were calculated

Our web collection on statistics for biologists contains articles on many of the points above.

Software and code

Policy information about availability of computer code

Data collection	The scRNA-seq and bulk RNA-seq data of this study have been deposited in the NCBI's BioProject under accession code PRJNA709641. Cell type was predicted by the web-based tool Enrichr (available at https://maayanlab.cloud/Enrichr/).
Data analysis	The raw data of scRNA-seq was processed by Drop-seq tools protocol (http://mccarrolllab.org/). Normalization was conducted by R package Seurat (version: 4.0.3). Data analysis was conducted through both python (version:3.8.8) and R (version:4.1.0). R package DEseq2(version:1.32.0) was used to pick differentially expressed genes (DEG) with q value < 0.05, fold change > 2 or < -2. Python package seaborn(version:0.11.1) was used to generate heatmaps.

For manuscripts utilizing custom algorithms or software that are central to the research but not yet described in published literature, software must be made available to editors and reviewers. We strongly encourage code deposition in a community repository (e.g. GitHub). See the Nature Portfolio guidelines for submitting code & software for further information.

Data

Policy information about availability of data

All manuscripts must include a data availability statement. This statement should provide the following information, where applicable:

- Accession codes, unique identifiers, or web links for publicly available datasets
- A description of any restrictions on data availability
- For clinical datasets or third party data, please ensure that the statement adheres to our policy

The scRNA-seq and bulk RNA-seq data of this study have been deposited in the NCBI's BioProject under accession code PRJNA709641.

Human research participants

Policy information about studies involving human research participants and Sex and Gender in Research.

Reporting on sex and gender	*Use the terms sex (biological attribute) and gender (shaped by social and cultural circumstances) carefully in order to avoid confusing both terms. Indicate if findings apply to only one sex or gender; describe whether sex and gender were considered in study design whether sex and/or gender was determined based on self-reporting or assigned and methods used. Provide in the source data disaggregated sex and gender data where this information has been collected, and consent has been obtained for sharing of individual-level data; provide overall numbers in this Reporting Summary. Please state if this information has not been collected. Report sex- and gender-based analyses where performed, justify reasons for lack of sex- and gender-based analysis.*
Population characteristics	*Describe the covariate-relevant population characteristics of the human research participants (e.g. age, genotypic information, past and current diagnosis and treatment categories). If you filled out the behavioural & social sciences study design questions and have nothing to add here, write "See above."*
Recruitment	*Describe how participants were recruited. Outline any potential self-selection bias or other biases that may be present and how these are likely to impact results.*
Ethics oversight	*Identify the organization(s) that approved the study protocol.*

Note that full information on the approval of the study protocol must also be provided in the manuscript.

Field-specific reporting

Please select the one below that is the best fit for your research. If you are not sure, read the appropriate sections before making your selection.

☒ Life sciences ☐ Behavioural & social sciences ☐ Ecological, evolutionary & environmental sciences

For a reference copy of the document with all sections, see nature.com/documents/nr-reporting-summary-flat.pdf

Life sciences study design

All studies must disclose on these points even when the disclosure is negative.

Sample size	In the section of "DE-MSCs modulate inflammatory responses in cell culture and mouse model", 50 male C57 mice were used to test the therapy effect of DE-MSCs on DSS-induced mice colitis. DSS+PBS group: 10 mice; DSS+PBS+DE-MSCs(FBS) group: 10 mice; DSS+PBS+DE-MSCs(CHIR) group: 10 mice; DSS+PBS+DE-MSCs(CHIR/SB) group: 10 mice; DSS+PBS+UC-MSCs group: 5 mice; Healthy control group: 5 mice.
Data exclusions	Not applicable.
Replication	Biological replicates for generation and characterization of mesenchymal stem cells (MSCs) were above 3 times, and all attempts at replication were successful.
Randomization	In the section of "DE-MSCs modulate inflammatory responses in cell culture and mouse model", all mice had similar age, and they were randomly allocated into each group, and the average weight per mouse in each group was 26±1g.
Blinding	In the section of "DE-MSCs modulate inflammatory responses in cell culture and mouse model", the assessment of therapy effect of DE-MSCs on DSS-induced mice colitis was a single blinding one.

Reporting for specific materials, systems and methods

We require information from authors about some types of materials, experimental systems and methods used in many studies. Here, indicate whether each material, system or method listed is relevant to your study. If you are not sure if a list item applies to your research, read the appropriate section before selecting a response.

Materials & experimental systems

n/a	Involved in the study	
☐	☒	Antibodies
☐	☒	Eukaryotic cell lines
☒	☐	Palaeontology and archaeology
☐	☒	Animals and other organisms
☒	☐	Clinical data
☒	☐	Dual use research of concern

Methods

n/a	Involved in the study	
☒	☐	ChIP-seq
☐	☒	Flow cytometry
☒	☐	MRI-based neuroimaging

Antibodies

Antibodies used: Anti-CD44 Mouse mAb (156-3C11) (1:1000, CST); Anti-CD73 antibody (ab54217) (1:1000, Abcam); Anti-CD105 antibody (ab11414) (1:1000, Abcam); Anti-PDGFR β antibody (ab69506) (1:1000, Abcam); Anti-CD45 antibody (ab10558) (1:1000, Abcam); Anti-Human CXCR4(CD184) (MHCXCR404) (1:1000, Life)

Validation: Anti-CD44 Mouse mAb: this primary antibody can be used for flow cytometric analysis for CD44, reference can be found by PMID 33156578, validation data can be found in this link https://www.cellsignal.com/products/primary-antibodies/cd44-156-3c11-mouse-mab/3570;

Anti-CD73 antibody: this primary antibody can be used for flow cytometric analysis for CD73, reference can be found by PMID 32705163, validation data can be found in this link https://www.abcam.cn/cd73-antibody-7g2-ab54217.html#lb;

Anti-CD105 antibody: this primary antibody can be used for flow cytometric analysis for CD105, reference can be found by PMID 33417281, validation data can be found in this link https://www.abcam.com/cd105-antibody-sn6-ab11414.html#lb;

Anti-PDGFR β antibody: this primary antibody can be used for flow cytometric analysis for PDGFR β, reference can be found by PMID 31611940, validation data can be found in this link https://www.abcam.com/pdgfr-beta-antibody-42g12-ab69506.html#lb;

Anti-Human CXCR4(CD184): this conjugated antibody can be used for flow cytometric analysis for CXCR4(CD184), reference can be found by PMID 30181898, validation data can be found in this link https://www.thermofisher.com/antibody/product/CXCR4-Antibody-clone-12G5-Monoclonal/MHCXCR404.

Eukaryotic cell lines

Policy information about cell lines and Sex and Gender in Research

Cell line source(s): H1 and H9 hESC lines (from WiCell Research Institute, Inc., Madison, WI, http://www.wicell.org). NL-1 iPSCs line (from NCRM)

Authentication: We have got the authentication from WiCell Research Institute to use H1 and H9 for scientific research purpose. We also have got the authentication from NCRM to use NL-1 for scientific research purpose.

Mycoplasma contamination: All cell lines were negative for mycoplasma contamination.

Commonly misidentified lines (See ICLAC register): *Name any commonly misidentified cell lines used in the study and provide a rationale for their use.*

Animals and other research organisms

Policy information about studies involving animals; ARRIVE guidelines recommended for reporting animal research, and Sex and Gender in Research

Laboratory animals: Species: mouse; Strain: C57BL/6J; Gender: male; Age: 8 weeks.

Wild animals: The study did not involve wild animals.

Reporting on sex: All the mice are male

Field-collected samples: The study did not involve samples collected from field.

Ethics oversight: The use of mice were approved by the University of Macau.

Note that full information on the approval of the study protocol must also be provided in the manuscript.

Flow Cytometry

Plots

Confirm that:

☒ The axis labels state the marker and fluorochrome used (e.g. CD4-FITC).

☒ The axis scales are clearly visible. Include numbers along axes only for bottom left plot of group (a 'group' is an analysis of identical markers).

☒ All plots are contour plots with outliers or pseudocolor plots.

☒ A numerical value for number of cells or percentage (with statistics) is provided.

Methodology

Sample preparation	MSCs were harvested by TrypLE (37°C, 5 min) and neutralized with 5% FBS. After washed with DPBS, cells were directly stained with primary antibody (0.5 hour, in room temperature) and then secondary antibody (0.5 hour, in room temperature and dark), washed 3 times, cells were resuspended with DPBS for flow cytometry analysis.
Instrument	Becton Dickinson C6 for flow cytometry; BD FACSAria-II for fluorescence-activated cell sorting (FACS)
Software	Software of Becton Dickinson C6; Software of BD FACSAria-II
Cell population abundance	For fluorescence-activated cell sorting (FACS), the sorted % of SOX17 positive cells was ~90%.
Gating strategy	Forward and side scatter density plots were used to identify live cell population and excluding debris, single parameter histograms for identifying cells with a particular marker expression were applied, and the positive vs. negative boundary was established by IgG isotype control.

☒ Tick this box to confirm that a figure exemplifying the gating strategy is provided in the Supplementary Information.
